# Effective and Secure Closure after Duodenal Endoscopic Submucosal Dissection: Combination of Endoscopic Ligation with O-Ring Closure and Over-the-Scope Clip

**DOI:** 10.3390/jcm12134238

**Published:** 2023-06-23

**Authors:** Kaho Nakatani, Hideki Kobara, Noriko Nishiyama, Shintaro Fujihara, Naoya Tada, Kazuhiro Koduka, Takanori Matsui, Taiga Chiyo, Nobuya Kobayashi, Tatsuo Yachida, Joji Tani, Asahiro Morishita, Hajime Isomoto, Tsutomu Masaki

**Affiliations:** 1 Department of Gastroenterology and Neurology, Faculty of Medicine, Kagawa University, Takamatsu 761-0793, Japan; kobara.hideki@kagawa-u.ac.jp (H.K.); nishiyama.noriko@kagawa-u.ac.jp (N.N.); fujihara.shintaro@kagawa-u.ac.jp (S.F.); tada.naoya@kagawa-u.ac.jp (N.T.); kozuka.kazuhiro@kagawa-u.ac.jp (K.K.); matsui.takanori@kagawa-u.ac.jp (T.M.); chiyo.taiga@kagawa-u.ac.jp (T.C.); kobayashi.nobuya.jn@kagawa-u.ac.jp (N.K.); yachida.tatsuo.fc@kagawa-u.ac.jp (T.Y.); tani.joji@kagawa-u.ac.jp (J.T.); morishita.asahiro@kagawa-u.ac.jp (A.M.); masaki.tsutomu@kagawa-u.ac.jp (T.M.); 2 Department of General Internal Medicine, Faculty of Medicine, Kagawa University, Takamatsu 761-0793, Japan; 3 Division of Gastroenterology and Nephrology, Faculty of Medicine, Tottori University, Tottori 683-8504, Japan; isomoto@tottori-u.ac.jp

**Keywords:** duodenal endoscopic submucosal dissection, over-the-scope clip, endoscopic ligation with O-ring closure

## Abstract

Duodenal endoscopic submucosal dissection (ESD) is associated with high incidences of intraoperative complications and delayed adverse events (AEs). Delayed AEs can be reduced by closing the post-ESD defects. We developed a new method of closure after duodenal ESD, combining endoscopic ligation with O-ring closure (E-LOC) with an over-the-scope clip (OTSC) (Band OTSC; B-OTSC). Here, we conducted a single-center, retrospective, observational study to investigate the efficacy and safety of the B-OTSC method for preventing delayed AEs in patients undergoing duodenal ESD. The study included nine patients with superficial nonpapillary duodenal epithelial tumors who underwent ESD and were closed with B-OTSC from February 2021 to February 2023. There were no delayed AEs (0%), the mean (± standard deviation) closure time was 53 ± 21.6 min, the complete closure rate was 100%, and the mean hospital stay was 7.8 ± 1.8 days. The sustained closure rates at postoperative days 3 and 7 were 88.9% and 88.9%, respectively. The historical analysis indicated a significant difference in cost between B-OTSC and conventional OTSC (*p* < 0.01). In conclusion, B-OTSC was a safe, secure, and cost-effective method of closure after duodenal ESD, even in patients with post-ESD defects of more than half the circumference.

## 1. Introduction

The detection rate of duodenal tumors has recently been increasing due to improved observational awareness and developments in endoscopic modalities [1]. The crude disease rate in Japan is 23.7 per million population [2], which is higher than the rate of 2.9–4.3 per million population in Europe and the United States [3,4,5]. However, there is currently no established endoscopic treatment strategy for duodenal tumors.

Superficial nonpapillary duodenal epithelial tumors (SNADETs) are considered to be a good indication for endoscopic treatment because of their low risk of lymph node metastasis from carcinoma in adenoma [6]. Underwater endoscopic mucosal resection (U-EMR) and EMR are often the treatments of choice for smaller tumors because of their relatively low complication rates. A large Japanese retrospective observational study by Kato et al. showed en bloc resection rates of 79.1%, 78.6%, 86.8%, and 94.8%, and delayed AE rates of 0.5%, 2.2%, 2.8%, and 6.8% in patients undergoing cold polypectomy, U-EMR, EMR, and ESD, respectively. The local recurrence rate was significantly lower in the ESD group compared with the non-ESD groups (*p* < 0.001) [7].

Duodenal ESD has a high incidence of intraoperative perforation, and delayed bleeding (DB) and perforation (DP) are also major adverse events (AEs) [8]. The incidence of delayed AEs is 6.8% [7], which is higher than that for gastric or colorectal ESD. The duodenal wall is thin and the submucosa contains large and dense blood vessels [9]. In addition, exposure of the post-ESD defects to bile and pancreatic juices increases the risk of delayed AEs [10]. In particular, larger lesions and areas distal to the papilla of Vater are associated with a higher incidence of delayed AEs [7]. Delayed AEs, DB, and DP were all significantly less common in patients who underwent closure compared with those without closure (6.4% vs. 20.9%, *p* < 0.0001; 5.5% vs. 17.5%, *p* < 0.0001; and 0.9% vs. 4.5%, *p* = 0.003, respectively) [8]. However, closing duodenal defects is difficult due to the narrow lumen and poor maneuverability. Furthermore, interestingly, there was no significant difference in the incidence of AEs between patients with partial closure and non-closure [11].

The over-the-scope clip (OTSC; Ovesco Endoscopy GmbH, Tübingen, Germany) method has demonstrated efficacy for closure after duodenal ESD [12]. It can provide durable complete closure with robust suture strength; however, it is relatively difficult to perform. In addition, OTSC closure of large post-ESD defects requires the use of a twin grasper (TG; Ovesco Endoscopy GmbH), which remains problematic in terms of cost.

Therefore, we developed a new closure method combining endoscopic ligation with O-ring closure (E-LOC) and OTSC (Band OTSC; B-OTSC) [13]. E-LOC closure, combining an O-ring and a clip, has been used to close post-ESD defects [14,15,16] and has also been applied to close gastrointestinal perforations [17,18]. In this study, we retrospectively investigated the efficacy and safety of the B-OTSC method for the closure of defects after duodenal ESD.

## 2. Materials and Methods

### 2.1. Study Design and Patients

This study was designed as a single-center, retrospective, observational study aimed at investigating the efficacy and safety of the B-OTSC method for preventing delayed AEs in patients undergoing duodenal ESD. All the procedures were conducted at a single institution (Kagawa University Hospital, Takamatsu, Japan), and the duodenal post-ESD defects were closed with B-OTSC according to our strategy. The study was approved by the Ethics Committee of Kagawa University Hospital (2022-139) and was registered in the Japan Registry of clinical Trials (JRCT No. 1060220111). All patients provided written informed consent to undergo the procedures. Nine consecutive patients with SNADETs >20 mm who were treated with ESD from February 2021 to February 2023 were enrolled in the study. In our strategy, we excluded lesions with a longitudinal diameter >40 mm in the axial direction of the intestine due to the difficulties encountered in closure. Lesions with incision lines near the papilla of Vater were also excluded because of the risk of acute pancreatitis due to closure. In addition, patients with lesions with suspected submucosal invasion and patients with severe comorbidities who would not tolerate surgery were also excluded. All the lesions were treated by two experienced ESD experts who had completed >1000 ESD procedures.

### 2.2. Preoperative Endoscopic Examination

The preoperative endoscopic examination was performed using a front-view high-resolution video endoscopy device (GIF-H290Z or GIF-XZ1200; Olympus, Tokyo, Japan) to evaluate the macroscopic type, size, location, and depth of the tumor. Depth was determined by endoscopic ultrasonography (UM-2 R system; Olympus, Tokyo, Japan). A hypoechoic area clearly infiltrating the third layer was considered indicative of submucosal invasion. Most patients in this study were referred from other hospitals, and preoperative biopsies had already been performed prior to referral to our hospital. If no biopsy had been performed at the referral hospital, it was performed at our hospital.

### 2.3. Duodenal ESD Procedure

All the procedures were performed in an operating room with the patient under general anesthesia. ESD was performed using an upper gastrointestinal endoscope (GIF-H290T; Olympus, Tokyo, Japan) with an elastic touch (F-010; TOP, Tokyo, Japan). All the procedures were performed with a high-frequency generator unit (VIO300D; Erbe Elektromedizin, Tübingen, Germany) and carbon dioxide insufflation.

After marking the boundary, a 1:1 solution of 0.4% hyaluronate sodium (MucoUp; Johnson and Johnson K.K., Tokyo, Japan) and glycerol (Chugai Pharmaceutical Co., Tokyo, Japan) mixed with diluted epinephrine (1:200,000) and indigo carmine was injected into the submucosal layer using a 25-G needle (Boston Scientific, Tokyo, Japan). 

Mucosal incision and submucosal dissection were then carried out using a DualKnife J (KD650Q; Olympus, Tokyo, Japan), ORISE ProKnife (Boston, Tokyo, Japan), or IT knife Nano (KD-612L; Olympus, Tokyo, Japan). The resection procedure was performed using either the counter traction [19] or submucosal tunneling method [20], depending on the lesion. Hemostasis of procedural bleeding was attempted using the knife or hemostatic forceps (Coagrasper FD-410LR; Olympus, Tokyo, Japan). We defined total procedure time as the resection time from injection to specimen retrieval.

### 2.4. B-OTSC Procedure

**Figure 1:**
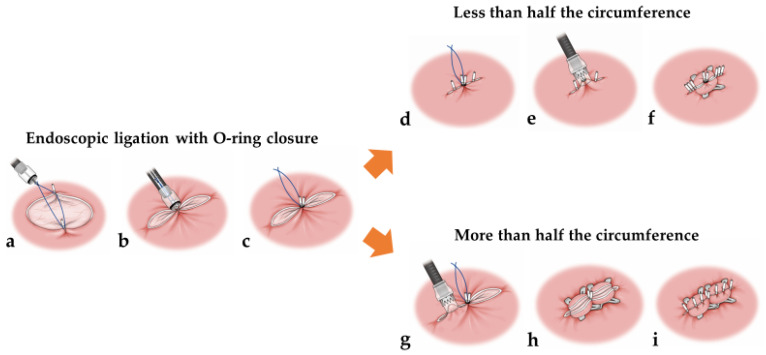
Schema showing the B-OTSC method. Endoscopic ligation with O-ring closure (**a**–**c**). A 2 cm diameter 3-0 surgical nylon loop was anchored at both edges in the center of the post-ESD defect using two hemoclips. Grasping forceps were then used to grasp the loop and pull it into the cap of an endoscopic variceal ligation device, and the deployed hemoclips were then pulled into the cap. An O-ring was fired around the hemoclips to shorten the post-ESD defect. Less than half the circumference (**d**–**f**). A 9 mm OTSC was fired using a simple suction technique at the E-LOC site after first adding hemoclips at both ends of the post-ESD defects. Additional hemoclips were used within the OTSC and the gap for complete closure. More than half the circumference (**g**–**i**). Two 9 mm OTSCs were placed on both sides of the E-LOC using a simple suction technique. Additional hemoclips were used within the OTSC and the gap for complete closure.

After specimen retrieval, closure of the mucosal defect was performed using the E-LOC method and OTSC (Video S1). The technical steps are summarized in Figure 1.

First, a 2 cm diameter 3-0 surgical nylon loop was anchored at both edges in the center of the post-ESD defect using two hemoclips (HX-610-090; Olympus, Tokyo, Japan or ROCC-D-26-195-C; MC medical, Tokyo, Japan). Grasping forceps were then used to grasp the loop and pull it into the cap of an endoscopic variceal ligation (EVL) device (MD-48720U; Sumius, Tokyo, Japan), and the deployed hemoclips were then pulled into the cap. An O-ring was fired around the hemoclips to shorten the post-ESD defect.

**Figure 2:**
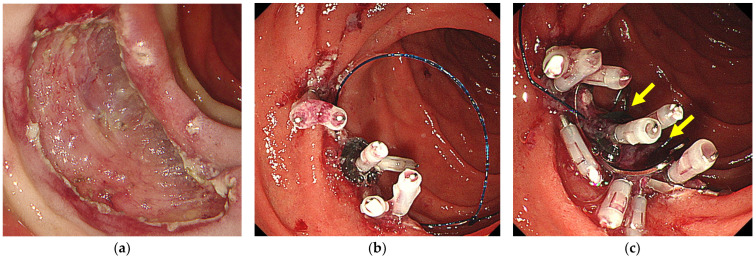
The B-OTSC method for post-ESD defects of less than half the circumference. (**a**) A post-ESD defect of less than half the circumference. (**b**) E-LOC shortens the post-ESD defect. (**c**) A 9 mm OTSC was placed on an E-LOC using a simple suction technique. Additional hemoclips were used for complete closure. The yellow arrows show the OTSC.

**Figure 3:**
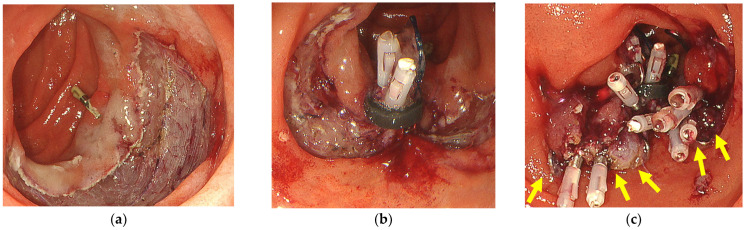
The B-OTSC method for post-ESD defects of more than half the circumference. (**a**) A post-ESD defect of more than half the circumference. (**b**) E-LOC shortens the post-ESD defect. (**c**) Two 9 mm OTSCs were placed on both sides of an E-LOC using a simple suction technique. Additional hemoclips were used for complete closure. The yellow arrows show the OTSCs.

If the post-ESD defect was less than half the circumference, a 9 mm OTSC (t-type; Ovesco Endoscopy GmbH, Tübingen, Germany) was fired using a simple suction technique at the E-LOC site after first adding hemoclips at both ends of the post-ESD defects because an OTSC could interfere with the application of additional hemoclips for complete closure (Figure 2a–c). Placement of an OTSC over the E-LOC simplifies the technique for OTSC placement. If the post-ESD defect was over half the circumference, two 9 mm OTSCs were placed on both sides of the E-LOC using a simple suction technique (Figure 3a–c). Additional hemoclips were used within the OTSC and the gap for complete closure.

### 2.5. Post-ESD Follow-Up

**Figure 4:**
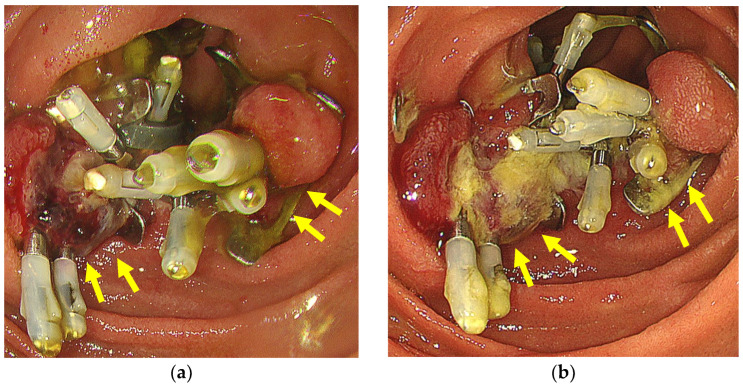
(**a**) Case showing complete and sustained closure on POD3. (**b**) Case showing complete and sustained closure on POD7. The yellow arrows show the OTSCs.

Oral intake of a liquid diet was resumed the day after ESD, which was then gradually replaced with solid foods on a daily basis. If symptoms such as fever or severe abdominal pain developed, an abdominal computed tomography (CT) scan was scheduled on an optional basis. Antibiotic prophylaxis was administered until postoperative day (POD) 1. A potassium competitive acid blocker (20 mg/day) was administered from POD0 until 2 months after ESD. Endoscopic follow-up after ESD was performed twice, on POD3 (Figure 4a) and POD7 (Figure 4b), respectively, and the patients were discharged from POD7.

### 2.6. Study Outcomes and Definition of AEs

The primary outcome was the prevalence of delayed AEs, defined as bleeding (hematemesis or melena, or a >2 g/dL decrease in serum hemoglobin) requiring endoscopic hemostasis, or perforations (diagnosed by radiography or CT, or intraperitoneal or retroperitoneal abscess formation) found after the end of the ESD procedure. DP was defined as a perforation diagnosed by radiography or CT, or intraperitoneal or retroperitoneal abscess formation found after the end of the ESD procedure.

The secondary outcomes included the complete closure rate, the defect closure time, sustained closure rates at POD3 and POD7, AEs related to B-OTSC, and the duration of hospitalization. Complete defect closure was defined as a successful closure covering >90% of the maximum wound length. The defect closure procedure time was defined as the duration between placement of the first and final hemoclips deployed on the defect. Sustained, partial, and unsustained closures were defined as maintenance of the wound length at >80%, 50% to <80%, and <50%, respectively. In an additional analysis, we compared the clinical efficacy and total costs of closure by B-OTSC with conventional OTSC (C-OTSC) using historical data in our hospital. The C-OTSC group compromised 11 consecutive patients who underwent duodenal ESD from January 2012 to July 2019, using TGs rather than E-LOC. We used TGs in all cases of C-OTSC. The shorter axis (S*n*) (cm) and longer axis (L*n*) of the ellipsoid resected specimen were measured after ESD. The ellipsoid resected area (A*n*) was defined as the area calculated by the following formula: A*n* (cm^2^) = π × S*n*/2 × L*n*/2 (π = 3.14) [*n* (B-OTSC) = 1–9] [*n* (C-OTSC) = 1–11]

The dissected area per minute during ESD (Da*n*) cm^2^/min = A*n*/DT*n* [*n* (B-OTSC) = 1–9] [*n* (C-OTSC) = 1–11]

Total cost was defined as the cost of all the OTSCs and EVL devices for the B-OTSC group and all the OTSCs and TG devices for the C-OTSC group. Additional clips were not included in the calculations because there was no significant difference between the groups (mean 9.6 clips in the B-OTSC group and 7.2 clips in the C-OTSC group). 

### 2.7. Statistical Analysis

The proportions and characteristics of the outcomes were presented as simple descriptive statistics. The categorical variables were expressed as absolute and relative frequencies. The differences between categorical variables were examined by Fisher’s exact test when required, and the continuous variables were compared using Mann–Whitney U test. *p* < 0.05 was considered statistically significant. All statistical analyses were conducted using JMP 15.1.0 (SAS Institute Inc., Cary, NC, USA).

## 3. Results

### 3.1. Patients and ESD Procedures

The patient and lesion characteristics and ESD outcomes are shown in Table 1. The mean (± standard deviation) age of the patients (six men and three women) was 67.2 ± 9.3 years. One patient was receiving antithrombotic medication (low-dose aspirin). The lesion was located in the second portion at the oral Vater in five patients and at the anal Vater in four patients. The macroscopic findings were elevated in eight patients and mixed in one patient. The lesion was less than half the circumference in six patients and over half the circumference in three patients. En bloc resection and R0 resection were achieved in all the patients, and there were no intraoperative complications. The mean tumor diameter was 26 ± 5.7 mm and the mean resected specimen diameter was 34.1 ± 6.4 mm. The mean procedure time was 119.4 ± 66.8 min. The pathological diagnosis was adenoma in five patients and tubular adenocarcinoma in four patients.

### 3.2. Outcomes of B-OTSC

Table 2 shows the outcomes of B-OTSC. There were no delayed AEs (0%), the complete closure rate was 100%, the mean closure time was 53 ± 21.6 min, the mean number of OTSCs was 1.4 ± 0.5, and the mean hospital stay was 7.8 ± 1.8 days. Furthermore, the sustained closure rates at PODs 3 and 7 were 88.9% and 88.9%, respectively. There were no AEs related to B-OTSC. The case of partially sustained closure on POD3 was due to the hemoclips being repelled.

In an additional analysis, we compared B-OTSC with conventional C-OTSC using historical data (Table 3). The mean resection diameter in the C-OTSC group was 27.3 ± 7.6 mm and that in the B-OTSC group was 34.1 ± 6.6 mm. There were no significant differences in the rates of complete closure or delayed AEs, even though the tumor diameter was larger in the B-OTSC group, but the speed of closure tended to be faster with B-OTSC than C-OTSC. B-OTSC reduced the number of OTSCs compared with C-OTSC, even though the B-OTSC group included three cases of post-ESD defects over half the circumference. B-OTSC was significantly cheaper than C-OTSC (mean $US 895 vs. $US 1755; *p* < 0.01).

## 4. Discussion

In this retrospective study, we treated nine patients with B-OTSC for the closure of post-ESD defects. Complete closure was achieved in all cases, with no postoperative AEs. The mean resection diameter was 34.1 ± 6.4 mm, and the study population included three patients with post-ESD defects over half the circumference. In addition, B-OTSC was significantly cheaper than C-OTSC. We identified three important findings regarding the B-OTSC method. First, we achieved complete and endurable closure of the post-ESD defects, even in cases where the lesion involved more than half the circumference. Second, B-OTSC was easier than C-OTSC because OTSCs could be placed with simple suction after shortening the post-ESD defects with E-LOC. Finally, B-OTSC offers a cost benefit compared to C-OTSC as it does not require TGs and reduces the number of OTSCs required.

Regarding AEs after duodenal ESD, DP and DB were previously reported to be less common in patients with defect closures compared to those without closure [21,22,23], and closure was reported to reduce complications by around 80% [21], with successful closure being an essential factor [24]. Various methods have been used to close post-ESD defects, including the string clip method [11], OTSC [25], and closing with an endoloop snare [23]. One study found no significant difference in the incidence of delayed AEs between patients with incomplete closure and non-closure (complete closure vs. incomplete closure vs. non-closure: 1.7% vs. 25.0% vs. 15.6%) [11]. In addition, they also reported that complete closure of the post-ESD defect was the only independent predictor of a reduced risk of DP in a multivariate analysis [11], indicating the importance of complete closure. Complete closure has also been shown to reduce the inflammatory response, measured as serum C-reactive protein, and to shorten fasting and hospital stay times [11]. Therefore, the complete closure of duodenal post-ESD defects is essential for reducing delayed AEs. However, the difficulties of endoscopic resection and closure are both increased in the duodenum due to its narrow lumen and poor endoscopic maneuverability. 

Mizutani et al. retrospectively analyzed the results of 698 duodenal endoscopic resection lesions and showed that a medial/anterior wall lesion location and a large lesion size were independent predictors of failure to close the mucosal defect, with a significantly lower incidence of complete closure for lesions located in the medial/anterior wall compared with the lateral/posterior wall (91% vs. 97%, *p* < 0.01). The incidence of complete closures was also significantly lower for lesions > 40 mm in size compared with smaller lesions (74% vs. 97%, *p* < 0.01). There was also a significant difference in the incidence of complete closures between lesions occupying ≤49% and ≥50% of the circumference (96% vs. 64%, respectively; *p* < 0.01) [26]. Thus, there is a need to develop a method for closing lesions that are located on the medial/anterior wall and at the supra-duodenal angle, lesions >40 mm, and lesions occupying ≥50% of the circumference. 

A high rate of complete closure of 90–98% has been reported for OTSC, compared with 67% for clip closure [25,27,28]. In terms of closure time, clip closure took longer than OTSC [12]. Furthermore, DP and DB were considered to be caused by a portion of the clip being repelled from the defect after closure, suggesting the need for a closure method with a stronger grasping force [24,29]. In the present study, the case of partially sustained closure on POD3 was due to the clips being repelled. Tashima et al. carried out a prospective interventional study of the effectiveness of OTSC and found that prophylactic defect closure with OTSC helped to reduce delayed AEs [27]. The complete closure rate with OTSC was 94% (47/50), but the difficulty of OTSC placement was strongly influenced by the location of the tumor and they considered that complete closure would be difficult for defects at the supra-duodenal angle. They also reported that large mucosal defects >30 mm in diameter increased the difficulty of OTSC placement. Complete closure using an OTSC and an endoloop snare has been reported, but a two-channel scope is required [30]. We believe that the current B-OTSC method allows for a high success rate of complete closure for post-ESD defects that are over half the circumference and in any location. In a large cohort comparing OTSC alone and OTSC with clips, the OTSC alone group had a significantly higher rate of DB (11.4% vs. 1.5%) [25]. Therefore, it seems likely that precoagulation of visible vessels and additional clip closure may be necessary for exposed defects, given that exposed defects reversed by OTSC are a cause of DB. 

Various studies have compared OTSC versus standard therapy (ST) for the treatment of nonvariceal upper gastrointestinal bleeding. A randomized controlled trial found a clinical success rate of 91.7% in the OTSC group compared with 73.1% in the ST group (*p* = 0.019), with significant differences in the occurrence of persistent bleeding between the groups (*p* = 0.027). All cases of bleeding were managed successfully by rescue therapy with OTSCs [31]. Schmidt et al. reported that OTSCs were more effective than ST in patients with recurrent bleeding from peptic ulcers. Persistent bleeding after per-protocol hemostasis was observed in 42.4% of patients in the ST group and 6.0% in the OTSC group (*p* = 0.001). Further bleeding occurred in 57.6% of the cases in the ST group and 15.2% in the OTSC group (absolute *p* = 0.001) [32]. In a subgroup analysis, OTSC as the primary therapy was associated with a significantly lower risk of rebleeding compared with ST (4% vs. 28.6%; *p* = 0.017) [33]. These results suggest that OTSCs might be recommended in patients who fail first hemostasis or those at high risk of rebleeding. A meta-analysis identified that the risk factors that independently predict rebleeding after endoscopic therapy include active bleeding at endoscopy, ulcer size, and bleeding from an ulcer located in the duodenal posterior wall or gastric high lesser curvature [34]. In addition to the duodenal posterior wall from the gastroduodenal artery, the first choice of OTSC is also considered for post-ESD duodenal ulcers with a large ulcer base, considering the risk of delayed perforation and bleeding.

In terms of the medical costs, C-OTSC and B-OTSC using OTSCs are more expensive than ST. However, the duodenal muscle layer is thin and there is a risk of perforation with hemostatic coagulation in cases of rebleeding, whereas hemoclips may be difficult to use in patients with chronic ulcers with fibrosis, and surgical salvage therapy after technical failure has been reported [32]. Therefore, it is preferable to use a closure method that can maintain durable complete closure to prevent rebleeding and delayed perforations. OTSC closure with robust suture strength is considered to be cost-effective in terms of rebleeding and delayed perforation.

The B-OTSC method has the advantage of simplicity. The post-ESD defect is shortened by E-LOC, and OTSC closure can be performed by a simple suction method without the use of a TG. Three defects were ≥50% of the circumference (33.3%), but all the defects (100%) were closed successfully. In addition, B-OTSC prevents the accidental suction of other organs outside the wall, which is considered a complication of OTSC. Only the duodenal wall can be closed by deploying an OTSC just on the E-LOC for lesions that are less than half the circumference. Furthermore, when an OTSC is applied to the slit-shaped defect area on either side of an E-LOC, the mucosa is in close proximity and is mainly closed in lesions that are greater than half the circumference. This technique is particularly useful for preventing injury of the pancreas for defects on the descending papillary side. However, B-OTSC should not be performed within 1 cm of the papilla of Vater in order to reduce the risk of pancreatitis. 

The closure line should be placed along the short axis direction due to the narrow lumen of the duodenum and the high risk of stenosis. Therefore, we excluded lesions with a longitudinal diameter > 40 mm in the axial direction of the intestine because it was difficult to close these with OTSCs in the short axis direction. These defects were also too long in the longitudinal direction to be closed by OTSCs and E-LOC. Thus, defects > 40 mm should be closed using the string clip method or poly-glycolic acid (PGA) sheets [35]; however, retention of the closure might be low.

One disadvantage of OTSC is that it is six to seven times more expensive than clip closure [12]; however, B-OTSC without TG is less expensive. In our additional historical analysis, even though the lesion size was larger in the B-OTSC group compared with the C-OTSC group, B-OTSC was significantly cheaper than C-OTSC due to the reduced number of OTSCs and the lack of TGs required. Shortening the defect with E-LOC also reduced the number of OTSCs.

Our study also had several limitations. First, it was a single-center, retrospective study with a small sample size. Second, the study did not include duodenal bulb cases and was biased at the site of the lesion. Therefore, further multicenter, prospective studies with larger sample sizes are needed to confirm the current results.

## 5. Conclusions

The B-OTSC method combining E-LOC and OTSC is a safe, effective, and cost-effective method for closing duodenal post-ESD defects, including those that are more than half the circumference.

## Figures and Tables

**Table 1:** Baseline characteristics and outcomes of ESD.

Characteristics	*n* = 9
Age, mean ± SD, year	67.2 (±9.3)
Antithrombotic therapy, *n* (%)	
LDA	1 (11.1)
Locations, *n* (%)	
First portion	0 (0)
Second portion (oral Vater)	5 (55.6)
Second portion (anal Vater)	4 (44.4)
Third portion	0 (0)
Macroscopic findings, *n* (%)	
Elevated	8 (88.9)
Depressed	0 (0)
Mixed	1 (11.1)
Occupied circumference, *n* (%)	
≤49%	6 (66.7)
≥50%	3 (33.3)
En bloc resection, *n* (%)	9 (100)
R0 resection rate, *n* (%)	9 (100)
Tumor diameter, mean ± SD (range), mm	26 (±5.7)
Resected specimen diameter, mean ± SD (range), mm	34.1 (±6.4)
Procedure time, mean ± SD (range), min	119.4 (±66.8)
Intraoperative complications, *n* (%)	0 (0)
Adenoma, *n* (%)	5 (55.6)
Tubular adenocarcinoma, *n* (%)	4 (44.4)

SD: standard deviation; LDA: low-dose aspirin; mixed: elevated and depressed.

**Table 2:** Outcomes of B-OTSC.

Variables	*n* = 9
Post-ESD complications (DB or DP) rate, *n* (%)	0 (0)
Complete closure rate, *n* (%)	9 (100)
Closure time of B-OTSC, mean ± SD (range), minutes	53 (±21.6)
Number of OTSC deployments, mean ± SD (range)	1.4 (±0.5)
Hospital stays after procedure, days, mean ± SD (range)	7.8 (±1.8)
AEs related to B-OTSC, *n* (%)	0 (0)
Sustained defect closure, *n* (%)	
On POD3	
Sustained, *n* (%)	8 (88.9)
Partially sustained, *n* (%)	1 (11.1)
Unsustained, *n* (%)	0 (0)
On POD7	
Sustained, *n* (%)	8 (88.9)
Partially sustained, *n* (%)	1 (11.1)
Unsustained, *n* (%)	0 (0)

SD: standard deviation; DP: delayed perforation; DB: delayed bleeding.

**Table 3:** Comparison of conventional OTSC (C-OTSC) and B-OTSC from historical data.

Patients	C-OTSC (*n* = 11)	B-OTSC (*n* = 9)	*p* Value
Age, mean ± SD, year	65 (±7.8)	67.2 (±9.3)	*p* = 0.31 *
Antithrombotic therapy, *n* (%)			*p* = 0.45 ^#^
LDA	0 (0)	1 (11.1)	
Tumor diameter, mean ± SD (range), mm	16.5 (±6.1)	26 (±5.7)	*p* < 0.01 *
Resected specimen diameter, mean ± SD (range), mm	27.3 (±7.6)	34.1 (±6.4)	*p* = 0.036 *
Locations, *n* (%)			*p* = 0.44 ^#^
First portion	1 (9.1)	0 (0)	
Second portion (oral Vater)	3 (27.3)	5 (55.6)	
Second portion (anal Vater)	6 (54.5)	4 (44.4)	
Third portion	1 (9.1)	0 (0)	
Occupied circumference, *n* (%)			*p* = 0.18 ^#^
≤49%	10 (90.9)	6 (66.7)	
≥50%	1 (9.1)	3 (33.3)	
Post-ESD complications (DB or DP) rate, n (%)	1 (9.1)	0 (0)	*p* = 0.35 ^#^
Closure time, mean ± SD (range), minutes	36.8 (±17)	53 (±21.6)	*p* = 0.087 *
The approximate ellipsoid resected area (A*n*) (cm^2^), mean ± SD (range)	469.6 (±294.3)	625.9 (±217.4)	*p* = 0.12 *
The closed area per minute (DA*n*) (cm^2^/min), mean ± SD (range)	13.9 (±8)	12.9 (±5.6)	*p* = 0.43 *
Complete closure rate, *n* (%)	11 (100)	9 (100)	*p* = 1 ^#^
Number of cases with 1/2/3 clips, *n* (%)	5(45.5)/3(27.3)/3(27.3)	6(66.7)/3(33.3)/0	*p* = 0.38 ^#^
Hospital stay after procedure, days, mean ± SD (range)	8.7 (±3.4)	7.8 (±1.8)	*p* = 0.41 *
Adverse events related to closure, *n* (%)	0 (0)	0 (0)	*p* = 1 ^#^
Cost (¥)/($), mean	237,000/1760	120,900/900	*p* < 0.01 *

LDA: low-dose aspirin; DP: delayed perforation; DB: delayed bleeding; (¥)/($): (Japanese yen)/(US dollar). * Mann–Whitney U test; ^#^ Fisher’s exact test.

## Data Availability

Data is not publicly available due to the protection of personal data and medical confidentiality.

## Supplementary Materials

The following supporting information can be downloaded at: https://www.mdpi.com/article/10.3390/jcm12134238/s1, Video S1. B-OTSC method for post-ESD defects of less than and more than half the circumference.
